# ELSA 2014 Cohort: Risk Factors Associated With Heavy Episodic Drinking Trajectories in Argentinean College Students

**DOI:** 10.3389/fnbeh.2020.00105

**Published:** 2020-06-17

**Authors:** Belén del Valle Vera, Angelina Pilatti, Ricardo Marcos Pautassi

**Affiliations:** ^1^Facultad de Psicología, Universidad Nacional de Córdoba, Córdoba, Argentina; ^2^Instituto de Investigaciones Psicológicas, IIPSI, UNC-CONICET, Córdoba, Argentina; ^3^Instituto de Investigación Médica M. y M. Ferreyra, INIMEC-CONICET, Universidad Nacional de Córdoba, Córdoba, Argentina

**Keywords:** heavy episodic drinking, trajectories, risk factors, college students, Argentina

## Abstract

Heavy episodic drinking (HED) is highly prevalent in college students. In Argentina, there is a notable lack of longitudinal studies examining drinking trajectories. The present study identified HED trajectories in Argentinean college students during the first 3 years of college (seven waves) and examined the association between risk factors for alcohol use and HED trajectories. The sample was composed of 1,240 college students [63.1% women, aged 18–25 years (*M* = 19.1 ± 1.7)] who completed at least three waves (the first data collection and ≥2 follow-ups). For 3 years, participants completed seven surveys that measured HED frequency, age of drinking onset, drunkenness occurrence, trait impulsivity, family history of alcohol abuse, stressful life events, and perceived peer’s drinking. Latent Class Growth Analysis (LCGA) and Multinomial Logistic Regression (MLR) were used to identify the pattern and number of HED trajectories and to explore which risk factors better distinguished between the trajectories, respectively. Six HED trajectories were identified: *Heavy Stable Frequency, Moderate Stable Frequency, Moderate Decreasing Frequency, Stable Infrequent, Decreasing Infrequent*, and *No-HED*. Younger age of drinking onset, alcohol intoxication, greater perception of peer drinking frequency and higher levels of impulsivity (i.e., sensation seeking, lack of premeditation, and positive urgency) increased the probability of belonging to the trajectories with more frequent HED. These trajectories partially coincide with those identified in studies from other cultures. Unlike previous studies, we did not find a trajectory with increasing/ascending HED frequency. This may be related to contextual/cultural variables unique to Argentina, like differences in the age when the peak in alcohol consumption is reached or the legal minimum age to buy alcoholic beverages in this country, and the idiosyncratic elements that characterize college life in Argentina. This work represents a step forward in the identification of risk factors differentiating between different HED trajectories, and help understand changes in alcohol use during college, in an understudied population.

## Introduction

Substance use is more frequent among emerging adults than in any other age group (Sedronar, 2017; Schulenberg et al., 2018). The transition from high school to college is a critical period for increments in alcohol use and alcohol-related negative consequences (Derefinko et al., 2016; Skidmore et al., 2016). Some evidence (Patrick and Terry-McElrath, 2017; Krieger et al., 2018; Schulenberg et al., 2018) suggests that alcohol use, and particularly heavy episodic drinking (HED), is more prevalent among college students than in their non-college peers.

HED is usually defined as the intake of ≥56/70 grams (women/men) of pure alcohol in a single, usually short, drinking occasion (Courtney and Polich, 2009). HED, which is highly prevalent (≈45–50%) among Argentinean emerging adults (Pilatti et al., 2014, 2017; Bravo et al., 2019) is associated with a wide variety of immediate and long-term negative consequences, such as academic problems, fainting, risky sexual behaviors, physical and sexual assaults, violent behavior, risk-taking and traffic accidents (Hingson, 2010; White and Hingson, 2013; Ferreira et al., 2014; Pilatti et al., 2016; Bravo et al., 2019). HED is also associated with an increased likelihood of developing alcohol (Kim et al., 2016; Cservenka and Brumback, 2017) or other substance use disorders (Dawson et al., 2010), and other mental health problems (Rehm, 2011). HED has also been shown to induce alterations in brain areas involved in drug reward, such as nucleus accumbens (Sousa et al., 2020).

Trajectory studies seek to explain behavior, including alcohol-related behavior, by accumulating data over time (Del Boca et al., 2004; Maggs and Schulenberg, 2004; Goudriaan et al., 2007). Most of the studies that examined alcohol use trajectories of college students found four types of trajectories (Greenbaum et al., 2005; Jackson et al., 2008; Sher et al., 2011; Derefinko et al., 2016): two stable (low/non-use or high use) and two dynamic (low/non-use that gradually increases over time, and other characterized by a high use that gradually decreases over time) trajectories. Some studies included a class of moderate alcohol use that remains stable over time (Ashenhurst et al., 2015; Derefinko et al., 2016) or a trajectory of low use punctuated by occasions of heavy drinking in specific periods, such as holidays (Greenbaum et al., 2005).

Successful interventions to reduce or delay alcohol drinking, or reduce the occurrence of HED and its negative consequences, should take advantage of identifying risk factors associated with these trajectories. Students engaged in heavy drinking trajectories exhibit, when compared to those of the other trajectories, higher trait impulsivity (Del Boca et al., 2004; Greenbaum et al., 2005; Jackson et al., 2008; Adams et al., 2013; Derefinko et al., 2016). Impulsivity is a multidimensional construct defined as the predisposition towards rapid and unplanned reactions, without considering the possible consequences (Moeller et al., 2001). Higher positive urgency, sensation seeking and lack of premeditation (i.e., facets of impulsivity, as suggested by the five-dimension UPPS-P impulsivity model; Lynam et al., 2007) are significant predictors of riskier drinking trajectories (Adams et al., 2013; Derefinko et al., 2016). Recent preclinical studies, in turn, suggest that the level of activation of cortico-mesencephalic tracts may predict greater vulnerability to the effects of binge drinking exposure on subsequent compulsive drinking (Siciliano et al., 2019).

Lower age of drinking onset and having experienced drunkenness (Casswell et al., 2002; Warner et al., 2007) are also significant predictors of riskier drinking trajectories. Strikingly, the odds of endorsing a heavy/risk drinking trajectory were significantly decreased with each additional year that drinking onset was delayed, and increased when participants reported they felt intoxicated during their first drinking episode (Warner et al., 2007). Peer factors, such as associating with peers who drink (Borsari and Carey, 2003), have been also related to engaging in heavy drinking trajectories (Chassin et al., 2002; Danielsson et al., 2010; Lee et al., 2012). Last but not least, a family history (FH+) of alcohol use disorders (Warner et al., 2007) and experiencing more stressful live events (Hanna et al., 2014) have been also regarded as genetic and environmental predictors, respectively, of riskier alcohol use trajectories.

The vast majority of studies on trajectories of alcohol use have been conducted with college students from the U.S. (Goudriaan et al., 2007; Sher et al., 2011) or Europe (Johnsson et al., 2008; Ståhlbrandt et al., 2010). In Argentina, the South American country where the present study was conducted, there is a notable lack of longitudinal epidemiological studies. Also, there are significant differences between the samples of college students employed in the previous studies and the Argentinean college students sampled in the present study. In fact, in Argentina, social organizations, such as fraternities or sororities, are non-existent and, different from the U.S. and some European countries, students cannot live on campus. Both features, availability of social organizations for students (Maggs et al., 2011; White and Hingson, 2013) and living on campus (Lorant et al., 2013) have been associated with heavier alcohol use. Additionally, in the U.S. the minimum legal age to buy alcohol is 21, whereas the minimum legal age is 18 in Argentina. Thus, Argentinean college students have legal access to alcohol during most of their college years. Noteworthy, in Argentina, different from the U.S. and most European countries, most college careers last between 5 and 6 years. Additionally, many careers require conducting final research to obtain a bachelor’s degree which, in turn, adds one more year. These variations could impact the prevalence and type of drinking trajectories.

The present study aimed to identify HED trajectories in college students since college entry and during the first 3 years of college (seven waves). In addition to identifying HED trajectories, we examined the relations between HED trajectories and risk factors linked with the development or maintenance of alcohol use, including trait impulsivity, age of drinking onset, alcohol intoxication, family history of alcohol abuse, stressful life events and perceived peer’s drinking. The study provides novel information, useful to understand changes in alcohol use during the college years of this understudied population, and, ultimately, should help in prevention or intervention efforts to reduce heavy alcohol use and its associated burden.

## Materials and Methods

### Participants

Participants were assessed seven times across 3 years: three times during the freshman year, twice during their sophomore year and twice during the third year of college. Four, out of the seven measures, were taken during the first semester of the academic year. The remaining three measures were taken during the second semester of the academic year. A total of 4,497 (57.8% women) college students completed the first assessment. However, only 1,977 could be contacted for the follow-ups (i.e., gave contact information). The sample analyzed in the present study (*n* = 1,240, 63.1% of women) comprises those who completed, at least, the first data collection, and ≥2 follow-ups. None of these measurements had to be necessarily consecutive. These participants had, at the commencement of the assessments, between 18 and 25 years old (*M* = 19.14, *SD* = 1.7). Demographic details can be found in Table 1. This sample of participants (*n* = 1,240) exhibited significant differences with those who dropped out of the study (*n* = 3,257). Specifically, those who dropped out from the study reported, compared to those who kept participating, significantly greater frequency of drinking (3.3 ± 2.9 vs. 2.9 ± 2.6 days, respectively; *t* = 4.738, *p* ≤ 0.001), significantly greater grams of alcohol consumed per drinking occasion (119.5 ± 98.7 vs. 112.3 ± 93.3, respectively; *t* = 2.272, *p* ≤ 0.05) and significantly greater alcohol-related negative consequences (8.7 ± 7.4 vs. 7.9 ± 6.8, respectively; *t* = 3.438, *p* ≤ 0.05). Moreover, those who dropped out were, at the beginning of the assessments, significantly younger (*t* = 2.09, *p* ≤ 0.05) than those who kept on participating (19.03 ± 1.6 vs. 19.14 ± 1.7 years, respectively), and men were more likely to be drop-outs group than women (*χ*^2^ = 22.364, *p* ≤ 0.001). Response rate across the follow-ups was: 73.2%, 57.5%, 53%, 50.8%, 43.9% and 34.3%.

**Table 1:** Description of sociodemographic variables for the total sample and as a function of sex.

	Total sample	Women (*n* = 782)	Men Age (*n* = 458)
Mean	19.14 ± 1.7	19.06 ± 1.68	19.29 ± 1.74
18–19	73.4	76.1	68.8
20–21	15	13.1	18.3
22–23	7.8	6.9	9.4
24–25	3.8	3.9	3.5
**Province of origin**			
Córdoba	71.9	72.9	70.1
Other	28.1	27.1	29.9
**Career**			
Economy	20.8	21.2	20.1
Psychology	13.9	18.3	6.3
Engineering	16.9	8.3	31.7
Medical sciences	11	13.5	6.8
Philosophy	9.5	9.6	9.9
Odontology	8.8	10.1	6.6
Other	19.1	19.1	18.6

*For categorical variables, values correspond to the percentage of participants placed in each category. For continuous variables, data are presented as means ± standard deviation*.

### Procedure

Thirteen departments of the National University of Cordoba (UNC, Argentina) received a brief invitation containing a description of the study and its aims. The National University of Cordoba (UNC, Argentina) began its activity in 1613 and is the oldest and one of the largest (approximately, 110,000 undergraduate students) universities of Argentina. Due to the central location of Cordoba City in Argentina, UNC attracts high-school graduates from different states of the country. The majority of the students were, however, from the Cordoba state and, particularly, from the city of Cordoba. These individuals belong to middle- and upper-middle-class families of large and medium-sized production farmers, professionals, and local merchants.

Ten departments accepted and authorized the research team to collect data in the classrooms. The researchers, in a face-to-face event, invited the students to participate in the study and explained its general aim, i.e., to better understand substance use behaviors during the college years, *via* a longitudinal study. Researchers also explained that participation involved completing a paper and pencil survey and several on-line follow-up surveys (six follow-ups during 3 years), which required providing contact information (e.g., email address). Students were informed about voluntary participation and anonymity. All procedures were approved by the university’s internal review board, and the protocol was reviewed by the National Agency for Promotion of Science and Technology.

### Measures

#### Alcohol Use

Following previous work (Pilatti et al., 2014), we used an *ad hoc* designed questionnaire to assess alcohol use in all data collections. We defined alcohol use as the consumption of at least one glass (i.e., 250 ml) of any alcoholic beverage. Participants indicated: (1) the two most consumed alcoholic beverages (e.g., beer, wine, vermouth, and so on); (2) usual frequency of alcohol use (i.e., from less than once per month to ≥4 times per week); and (3) number of glasses (a standard size of glass was provided) consumed per drinking occasion (i.e., from 1 glass to ≥14 glasses). Those who have never drunk alcohol or who abstained from drinking within the previous year had an option to indicate so. Based on known alcoholic contents in each alcoholic beverage, answers to questions 1 and 3 were used to calculate the grams of alcohol consumed per drinking occasion. To estimate the total volume of alcohol consumed within a month, we calculated the product of usual frequency (i.e., the number of drinking days within a month) by the grams of alcohol consumed per drinking occasion. Both grams per drinking occasion and total volume were considered continuous variables. Participants also indicated usual frequency (i.e., from less than once per month to ≥4 times per week) of HED (i.e., ≥4/5 standard drinks (women/men) in a single drinking occasion (as defined by the Ministerio de Salud Argentino, 2012). The questionnaire provided, to facilitate the identification of HED, examples of the quantity of alcoholic beverages that corresponds to a heavy drinking episode (e.g., 6/7 glasses of beer or 4/5 glasses of wine for women and men respectively). Previous work examined the correspondence between estimates of alcohol consumption obtained with this *ad hoc* retrospective questionnaire with those obtained through an alcohol consumption diary (Pilatti et al., submitted). Results showed a large correspondence between those estimates, providing evidence on the validity of these retrospective estimates of alcohol consumption. Finally, students were asked to report: (i) the age they first drank one standard drink or more of any alcoholic beverage; and (ii) the occurrence of drunkenness episodes in their lifetime.

#### Alcohol-Related Negative Consequences

We used the Spanish version (S-YAACQ; Pilatti et al., 2016) of the Young Adult Alcohol Consequences Questionnaire (YAACQ; Read et al., 2006) to measure 48 alcohol-related negative consequences. Participants indicated whether or not they had experienced each consequence within each period. In the first data collection the timeframe was the previous year, and in the remaining surveys was the previous 3 months. The total score reflects the total number of consequences that the individual has experienced and, thus, was considered a continuous variable. The internal consistency, estimated with the tetrachoric correlation coefficient (Ledesma et al., 2011), was 0.90. Although this questionnaire was included in the first, third, fourth, sixth, and seventh data collection; the results described in the present study correspond to the first and last data collection.

#### Impulsivity

We used the Spanish version (Verdejo-García et al., 2010) of the UPPS-P, which features 59 items to measure five dimensions of impulsivity: Positive Urgency (*α* = 0.90), Negative urgency (*α* = 0.84), Lack of Premeditation (*α* = 0.83), Lack of Perseverance (*α* = 0.78) and Sensation Seeking (*α* = 0.84). The items are scored on a 4-point scale, ranging from 1 (strongly agree) to 4 (strongly disagree). Items of each sub-scale are summed up and higher scores indicate higher impulsivity levels. Scores in each sub-scale are treated as continuous variables. Impulsivity was measured during the second data collection.

#### Stressful Life Events

The Inventory of Stressful Vital Events (Inventario de Acontecimientos Vitales Estresantes, AVE, Oliva et al., 2008) was used. The inventory consists of 29 items that evaluate stressful life events experienced during the last year, in different contexts (e.g., family, academic or work environment, peer group, etc.). Affirmative responses are summed; thus, the total score reflects the total number of stressful events that the individual has experienced. The total score is treated as a continuous variable. The internal consistency, estimated with the tetrachoric correlation coefficient (Ledesma et al., 2011), was 0.78. This questionnaire was included during the second data collection.

#### Descriptive Social Norms for Alcohol Use

Based on Pilatti et al. (2013), we asked the perceived frequency of alcohol use for the closest female and male friend (i.e., from less than once per month to ≥4 times per week). Those whose friends did not drink alcohol had an option to indicate so. This question allowed calculating the friend’s number of days with alcohol consumption per month (continuous variable). Descriptive social norms for alcohol use were measured during the first data collection.

#### Family History of Alcohol Use Disorders

Following LaBrie et al. (2010), participants reported if a first (mother or father), second (siblings or grandparents) or third-degree relative (uncles) has or has had a history of alcohol abuse that derived, or could have derived, in treatment. Those reporting a family member with those characteristics were considered FH+ and those who did not were considered FH−. Family history of alcohol use disorders was measured during the first data collection.

### Analysis Strategy

Latent Class Growth Analysis (LCGA) was performed to identify the HED trajectories that best fit the data. LCGA allows identifying qualitatively different groups or classes (Muthén and Muthén, 2000) that exhibit prototypical patterns of change over time, for an outcome of interest. Thus, individuals within each class differ in the level of the variable of interest (e.g., frequency of HED) at the beginning of the time window examined (intercept) and at the pattern of change (slope) over time (McArdle and Nesselroade, 2003). LCGA allows individuals with missing data to still contribute to the estimation of model parameters. Trajectories were estimated using seven data points per individual representing the usual frequency of HED (0 = no HED, 1 = less than monthly, 2 = monthly, 3 = two to three times per month, 4 = weekly, 5 = two times per week and 6 = three or more times per week).

LCGA models with two to seven latent classes were sequentially tested to determine the best fitting and most parsimonious model. The best-fitting model was selected based on interpretability and theoretical relevance of the classes, and on several statistical criteria: Akaike information criterion (AIC; Akaike, 1987), Bayesian information criterion (BIC; Sclove, 1987), entropy values and Lo-Mendell-Rubin adjusted likelihood ratio tests (LMR-LRT, Lo et al., 2001). To ensure that the latent classes capture a meaningful portion of the sample, each class had to include at least 5% of the sample (Nagin and Tremblay, 2005). We then applied one-way ANOVAs to several alcohol use indicators (grams consumed per drinking occasion, the volume of alcohol consumed within a month and negative consequences experienced—calculated only among last-year drinkers), with the identified trajectories as between factor, at the beginning (Time 1) and end (Time 7) of the examined period.

Once participants had been assigned to a trajectory group, multinomial logistic regression (MLR) was used to identify which variables predicted group membership. MLR explores how a set of variables distinguishes between different categories of a categorical dependent measure (i.e., the HED trajectories). The independent variables, all measured during the first or second data collection, were the age of drinking onset, alcohol intoxication (yes/no), the five dimensions of trait impulsivity (according to UPPS-P), best female and male friend perceived drinking frequency, stressful life events experienced and family history of alcohol use disorders (yes/no). The odds ratios (ORs) and 95% confidence intervals (CI) were estimated. The ANOVAs and MLR were performed using SPSS 20.0, whereas LCGA was run in Mplus version 6.12. Alpha value was set at 0.05. The data that support the findings of this study are available from the corresponding author upon reasonable request.

## Results

### Identification and Description of HED Trajectories

**Figure 1:**
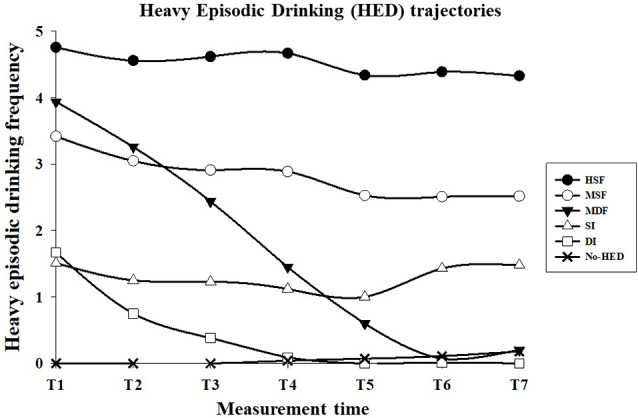
Trajectories of heavy episodic drinking (HED) frequency since college entry and during the first 3 years of college. Frequency of HED: 0 = no HED, 1 = less than monthly, 2 = monthly, 3 = 2–3 times per month, 4 = weekly, 5 = 2 times per week, and 6 = 3 or more times per week. HSF, Heavy Stable Frequency, 9%; MSF, Moderate Stable Frequency, 30%; MDF, Moderate Decreasing Frequency, 7%; SI, Stable Infrequent, 22%; DI, Decreasing Infrequent, 13%; No-HED, without HED, 19%.

The six-class model was selected. The BIC, AIC, LMR-LRT *p*-value, and entropy values for the different models are shown in Table 2. The BIC and AIC values continued to decrease with an increasing number of classes. However, the entropy value, the posterior probabilities, the LMR-LRT adjustment index, and the theoretical relevance of the classes favored the six-class model. Figure 1 shows the adjusted growth curves for the six HED trajectories identified and Table 3 shows the precision of latent class assignment, the estimated class proportions, and the Intercept and Slope values for each class.

**Table 2:** Fit statistics for the different latent growth models.

Number of classes	AIC	BIC	aBIC	Entrophy	LMR-LRT *p*-value
2	19,387.373	19,438.602	19,406.837	0.835	0.000
3	18,724.566	18,791.163	18,749.869	0.826	0.001
4	18,505.477	18,587.443	18,536.620	0.767	0.000
5	18,394.378	18,491.713	18,431.360	0.758	0.004
**6**	**18,319.658**	**18,432.361**	**18,362.480**	**0.740**	**0.000**
7	18,290.236	18,418.308	18,338.897	0.700	0.133

*AIC, Akaike Information Criterion; BIC, Bayesian Information Criterion; aBIC, sample-size adjusted Bayesian Information Criterion; LMR-LRT, Lo-Mendell-Rubin adjusted LRT test. The selected model is indicated in bold font*.

**Table 3:** Growth factor parameter estimates and posterior probabilities for the 6-Class model.

Classes	Class proportion	Classification accuracy	Intercept	Slope
Class 1	0.13	0.80	−3.489	−1.003***
Class 2	0.09	0.89	1.599	−0.138*
Class 3	0.30	0.83	−0.901	−0.201***
Class 4	0.19	0.86	−9.625	0.580**
Class 5	0.22	0.79	−3.463	−0.055
Class 6	0.07	0.70	0.000	−1.130***

*p < 0.05, **p < 0.01, ***p < 0.001.

Based on the shape of the growth curves, the classes were labeled: (1) *Heavy Stable Frequency* -HSF- (*n* = 116, 9.4%) included those who drank the heaviest (i.e., reported HED almost twice a week) consistently over the 3 years; (2) *Moderate Stable Frequency* -MSF- (*n* = 373, 30.1%) included those who had a moderate HED (i.e., 2–4 per month) across the 3 years; (3) *Moderate Decreasing Frequency* -MDF- (*n* = 88, 7.1%) included those who began the study with moderate levels of drinking (i.e., HED once per week), but declined as the study progressed; (4) *Stable Infrequent* -SI- (*n* = 274, 22.1%) included those who had HED less than monthly consistently over the 3 years; (5) *Decreasing Infrequent* -DI- (*n* = 156, 12.6) included those who began the study having heavy drinking episodes less than monthly, but declined as the study progressed; and (6) *No-HED* (*n* = 233, 18.8%) included those that did no exhibit HED over the timeframe examined.

#### Differences in Alcohol Use Between the HED Trajectories

At Time 1, the HED trajectories significantly differed in the grams of alcohol consumed per drinking occasion (*F*_(1236)_ = 179.42, *p* < 0.001), the monthly volume of alcohol consumed (*F*_(1239)_ = 190.64, *p* < 0.001) and the number of negative consequences reported (*F*_(1147)_ = 102.44, *p* < 0.001). Mean values for each class are presented in Table 4. The *post hoc* analysis revealed, for both grams per drinking occasion and alcohol-related negative consequences, significantly greater mean values in the *Heavy Stable Frequency* class when compared to the *Moderate Stable Frequency* or the *Moderate Decreasing Frequency* class. The latter two classes scored significantly higher than the *Stable Infrequent/Decreasing Infrequent* classes, which in turn scored significantly higher than the *No-HED* class. For the volume of alcohol consumed within a month, the *post hoc* analyses revealed greater mean values in the *Heavy Stable Frequency* than in the *Moderate Decreasing Frequency* class. The latter class scored significantly higher than the *Moderate Stable Frequency* class, which in turn scored significantly higher than the *Stable Infrequent* and *Decreasing Infrequent* classes. The *No-HED* scored significantly lower than the rest of the classes.

**Table 4:** Alcohol use indicators as a function of class membership during Time 1 and Time 7.

	HSF		MSF		MDF		SI		DI		No-HED	
	Time 1	Time 7	Time 1	Time 7	Time 1	Time 7	Time 1	Time 7	Time 1	Time 7	Time 1	Time 7
Grams per drinking occasion	219.96 ± 10.02	146.26 ± 10.01	156.38 ± 4.47	102.4 ± 4.25	159.82 ± 9.18	44.77 ± 5.78	88.32 ± 3.67	68.78 ± 3.12	77.88 ± 3.91	28.15 ± 2.55	21.24 ± 1.37	22.11 ± 2.27
Usual Frequency	6.92 ± 0.3	7.07 ± 0.54	3.65 ± 0.12	3.32 ± 0.19	4.22 ± 0.29	2.27 ± 0.45	1.92 ± 0.08	2.61 ± 0.17	2.01 ± 0.14	1.52 ± 0.24	0.81 ± 0.05	1.01 ± 0.12
Volume of alcohol	17,51.87 ± 122.83	968.49 ± 81.92	620.21 ± 27.11	349.47 ± 22.06	809.89 ± 80.57	115.49 ± 18.72	194.08 ± 10.37	186.18 ± 13.46	195.94 ± 18.82	70.53 ± 47.56	33.92 ± 3.18	44.89 ± 6.59
Drunkenness episodes	0.53 ± 0.07	1.23 ± 0.31	0.26 ± 0.02	0.58 ± 0.08	0.25 ± 0.05	0.21 ± 0.09	139 ± 0.02	0.4 ± 0.08	0.06 ± 0.02	0.05 ± 0.03	—	0.13 ± 0.04
Negative consequences	14.51 ± 0.74	12.43 ± 1.31	11.22 ± 0.33	7.06 ± 0.44	10.1 ± 0.66	4.93 ± 1.46	6.75 ± 0.30	5.38 ± 0.47	5.89 ± 0.4	1.64 ± 0.29	1.78 ± 0.21	1.47 ± 0.38

*Data are presented as means ± standard errors of the mean. HSF, Heavy Stable Frequency; MSF, Moderate Stable Frequency; MDF, Moderate Decreasing Frequency; SI, Stable Infrequent; DI, Decreasing Infrequent; No-HED, without Heavy Episodic Drinking*.

At Time 7, the grams of alcohol consumed per drinking occasion (*F*_(633)_ = 89.57, *p* < 0.001), the monthly volume of alcohol consumed (*F*_(633)_ = 113.69, *p* < 0.001) and the negative consequences (*F*_(581)_ = 28.68, *p* < 0.001) significantly differed as a function of the class. For grams of alcohol consumed per drinking occasion, the pattern of observed differences were as follows: *Heavy Stable Frequency* > *Moderate Stable Frequency* > *Stable Infrequent* > *Moderate Decreasing Frequency/Decreasing Infrequent/No-HED*. For the volume of alcohol consumed within a month, the *post hoc* analysis revealed that *Heavy Stable Frequency* > *Moderate Stable Frequency* > *Stable Infrequent* > *Decreasing Infrequent/No-HED*. The *Moderate Decreasing Frequency* trajectory was significantly different from all the classes, except for the *Stable Infrequent* and *Decreasing Infrequent/No-HED* trajectories. The *post hoc* tests conducted for the number of alcohol-related negative consequences showed greater mean values in the *Heavy Stable Frequency* class when compared to the *Moderate Stable Frequency* or the *Stable Infrequent* classes. The latter two classes scored significantly higher than the *Decreasing Infrequent* and *No-HED* classes. *Moderate Decreasing Frequency* trajectory located in an intermediate position between the *Stable Infrequent* and *Decreasing Infrequent* trajectories, without showing significant differences from these trajectories but significantly differing from the others (mean values presented at Table 4).

#### Predictors of HED Trajectories

The Deviance criterion ($\chi_{\left(4,952\right)}^{2}$ = 2809.73, *p* = 1.00) indicated a good model fit (discrimination among groups) based on 11 variables. The model explained 43% of the variance (Nagelkerke’s Pseudo *R*^2^ = 0.435). Likelihood ratio tests showed six of the variables were significant predictors of class membership (age of drinking onset, drinking intoxication, lack of premeditation -PREM-, positive urgency -PU-, sensation-seeking -SS- and perceived drinking frequency of the best same-sex friend). Correct classification rates using these variables were 76.8% for *Moderate Stable Frequency*, 68.2% for *No-HED*, 27.3% for *Stable Infrequent*, 4.5%, and 21.7% for *Decreasing Infrequent* and *Heavy Stable Frequency*, respectively. There was no correct classification for the *Moderate Decreasing Frequency* group. The overall correct classification rate was 44.3%.

Table 5 reports ORs and 95% CIs for the variables included in the model, comparing the *No-HED* trajectory vs. the others. Younger age of drinking onset (OR = 0.59 and OR = 0.67 for *Heavy Stable Frequency* and *Moderate Stable Frequency* trajectories, respectively), alcohol intoxication (OR = 156.55 and OR = 43.43 for *Heavy Stable Frequency* and *Moderate Stable Frequency* trajectories, respectively), greater sensation seeking (OR = 1.06 and OR = 1.05 for *Heavy Stable Frequency* and *Moderate Stable Frequency* trajectories, respectively), greater positive urgency (OR = 1.09 and OR = 1.07 for *Heavy Stable Frequency* and *Moderate Stable Frequency* trajectories, respectively) and a perception of a greater frequency of alcohol use among same-sex peers (OR = 1.33 and OR = 1.14 for *Heavy Stable Frequency* and *Moderate Stable Frequency* trajectories, respectively) predicted membership to the trajectories with greater HED, compared to the *No-HED* trajectory. Considering the *Moderate Decreasing Frequency* trajectory as the comparison group, alcohol intoxication was significantly associated to the *Heavy Stable Frequency* (OR = 18.29) and *Moderate Stable Frequency* (OR = 5.07) trajectories, and lower levels of lack of premeditation were associated to the *Decreasing Infrequent* (OR = 0.91), *Stable Infrequent* (OR = 0.92) and *No-HED* (OR = 0.92) trajectories (results in Table 6).

**Table 5:** Multinomial logistic regressions comparing No-HED trajectory vs. the others: odds ratio estimates.

	Heavy Stable Frequency vs. No-HED			Decreasing Infrequent vs. No-HED			Stable Infrequent vs. No-HED			Moderate Decreasing Frequency vs. No-HED			Moderate Stable Frequency vs. No-HED		
	OR	95% CI		OR	95% CI		OR	95% CI		OR	95% CI		OR	95% CI	
		LB	UB		LB	UB		LB	UB		LB	UB		LB	UB
Age Onset	**0.59**	0.47	0.73	0.90	0.77	1.06	**0.78**	0.67	0.91	**0.67**	0.54	0.84	**0.67**	0.57	0.74
Intoxication	**156.55**	19.24	1,273.90	**5.75**	3.45	9.59	**12.36**	7.39	20.69	**8.56**	3.95	18.54	**43.43**	21.44	87.98
UN	1.02	0.96	1.08	0.96	0.92	1.01	1.01	0.96	1.06	1.00	0.94	1.06	0.99	0.95	1.04
PREM	1.06	0.99	1.14	0.98	0.93	1.04	1.00	0.94	1.05	**1.08**	1.01	1.16	1.04	0.98	1.10
PERSEV	0.99	0.92	1.06	1.03	0.97	1.09	1.00	0.95	1.06	1.01	0.94	1.09	0.98	0.93	1.04
SS	**1.06**	1.02	1.11	1.00	0.96	1.03	1.02	0.99	1.06	1.03	0.98	1.07	**1.05**	1.01	1.08
PU	**1.09**	1.03	1.15	**1.06**	1.00	1.11	1.02	0.97	1.07	1.05	0.99	1.11	**1.07**	1.02	1.12
Same-sex drinking	**1.33**	1.18	1.47	1.05	0.94	1.17	1.03	0.93	1.14	1.12	0.99	1.27	**1.14**	1.03	1.26
Opposite-sex drinking	1.02	0.92	1.11	0.97	0.89	1.06	0.99	0.91	1.07	1.00	0.90	1.11	0.95	0.88	1.04
FH+	0.78	0.40	1.52	0.73	0.43	1.26	0.60	0.36	0.99	0.86	0.45	1.68	0.50	0.30	0.84
Stressful events	0.97	0.89	1.05	0.97	0.91	1.04	0.99	0.94	1.05	1.00	0.92	1.08	0.99	0.93	1.05

*OR, odds ratios; CI, confidence intervals; LB, lower bound; UB, upper bound; UN, negative urgency; PREM, lack of premeditation; PERS, lack of perseverance; SS, sensation seeking; PU, positive urgency; FH+, family history of alcohol abuse. Significant *odd ratios* are indicated in bold font*.

**Table 6:** Multinomial logistic regressions comparing the Moderate Decreasing Frequency trajectory vs. the others: odds ratio estimates.

	Heavy Stable Frequency vs. MDF			Decreasing Infrequent vs. MDF			Stable Infrequent vs. MDF			Moderate Stable Frequency vs. MDF		
	OR	95% CI		OR	95% CI		OR	95% CI		OR	95% CI	
		LB	UB		LB	UB		LB	UB		LB	UB
Age Onset	0.87	0.68	1.11	**1.34**	1.08	1.67	1.16	0.95	1.42	0.99	0.81	1.21
Intoxication	**18.29**	2.09	159.67	0.67	0.30	1.51	1.44	0.65	3.22	**5.07**	2.02	12.75
UN	1.02	0.96	1.08	0.96	0.91	1.02	1.01	0.96	1.07	0.99	0.94	1.04
PREM	0.98	0.91	1.05	**0.91**	0.85	0.98	**0.92**	0.87	0.98	0.96	0.91	1.02
PERSEV	0.98	0.91	1.06	1.02	0.95	1.09	0.99	0.93	1.06	0.98	0.92	1.04
SS	1.04	0.99	1.09	0.97	0.93	1.01	1.00	0.96	1.04	1.02	0.98	1.06
PU	1.04	0.99	1.10	1.01	0.96	1.07	0.97	0.93	1.02	1.02	0.97	1.07
Same-sex drinking	**1.18**	1.05	1.33	0.93	0.82	1.06	0.91	0.81	1.02	1.01	0.91	1.13
Opposite-sex drinking	1.01	0.91	1.13	0.97	0.87	1.08	0.99	0.90	1.09	0.95	0.87	1.04
FH+	0.90	0.44	1.82	0.85	0.44	1.64	0.69	0.38	1.26	0.58	0.32	1.04
Stressful events	0.97	0.89	1.06	0.98	0.90	1.06	1.00	0.93	1.07	0.99	0.93	1.06

*OR, odds ratios; CI, confidence intervals; LB, lower bound; UB, upper bound; UN, negative urgency; PREM, lack of premeditation; PERS, lack of perseverance; SS, sensation seeking; PU, positive urgency; FH+, family history of alcohol abuse. Significant odd ratios are indicated in bold font*.

## Discussion

This study identified, in the understudied population of Argentinean college students, six HED trajectories during the first 3 years of college: a trajectory with frequent and stable HED (*Heavy Stable Frequency*); two trajectories with moderate HED at the beginning of the examined period, one that remained stable over time (*Moderate Stable Frequency*) and one with a marked decrease in HED frequency over time *(Moderate Decreasing Frequency*); two trajectories with infrequent HED, one that remained stable over time (*Stable Infrequent*) and one with a marked decrease in HED frequency over time (*Decreasing Infrequent*); and, finally, a trajectory with no HED or very low frequency (*No-HED*). These trajectories showed differences in alcohol use. Notably, the *Heavy Stable Frequency* and *No-HED* trajectories seemed to present the greatest and lowest risk, since their members exhibited the greatest and lowest levels of alcohol consumption and alcohol-related negative consequences, respectively.

The identified trajectories partially coincide with those found in studies from other cultures. Similar to other studies (Tucker et al., 2003; Goudriaan et al., 2007; Jackson et al., 2008; Sher et al., 2011; Ashenhurst et al., 2015), we identified the high and stable frequency trajectory, the low and stable frequency trajectory, a near-zero frequency trajectory, a moderate and stable frequency trajectory and two decreasing frequency trajectories that had different levels of alcohol consumption at the beginning of the study.

Unlike previous studies (Chassin et al., 2002; Jackson et al., 2008; Sher et al., 2011; Ashenhurst et al., 2015), we did not find a trajectory with increasing HED frequency. This could relate to the fact that alcohol consumption was measured at a time when all students had already reached their peak in alcohol use. The average age of the students at the beginning of this study was 19.14 ± 1.7 years old, roughly one more year than those college students in the studies that identified an increasing alcohol use trajectory (Greenbaum et al., 2005; Sher et al., 2011; Ashenhurst et al., 2015). Also, in Argentina, the minimum legal age to buy alcohol is 18 years old, which means that the participants of our study had already legal access to buy alcohol at the beginning of the measurement. This, in turn, may affect the age when the peak in alcohol consumption is achieved. Supporting this idea, it has been found that, in the United Kingdom, where the legal age to buy alcohol is 18 years old, the peak in alcohol consumption occurs between 18–19 years old (Bewick et al., 2008), while in the U.S., where the legal age to buy alcohol is 21 years old, it has been usually registered between 21–22 years of age (Chen and Jacobson, 2012; Jager et al., 2015; Patrick et al., 2016). Finally, we need to remember that Argentinean students cannot affiliate to fraternities or sororities nor live on campus, all of which has been associated with heavier alcohol use (Barry, 2007; Maggs et al., 2011; Lorant et al., 2013; White and Hingson, 2013). Instead, during their college years, most Argentinean college students continue living with their families under parental supervision, which could discourage heavy alcohol use (Evans-Polce et al., 2017; Patrick and Terry-McElrath, 2017).

Regarding risk factors that discriminated between the identified trajectories, the results showed that those with a younger age of drinking onset and whoever felt intoxicated had a markedly higher probability of belonging to the *Heavy Stable Frequency* and *Moderate Stable Frequency* trajectories than to the *No-HED* trajectory. These results coincide with those found in cross-sectional and prospective studies (Kuntsche et al., 2013; Moss et al., 2014; Jackson et al., 2015; Asbridge et al., 2016), where a lower age of drinking onset and alcohol intoxication has been associated with increased alcohol consumption, and also with trajectory studies in which early drinking onset increases the probability of belonging to a stable frequent drinking trajectory (Casswell et al., 2002). It should be noted that the effect of alcohol intoxication was greater than the effect of drinking onset. The present findings extend the conclusions of our previous analysis of the ELSA 2014 study (using data from the first-wave, Vera et al., 2020) where we examined the interactive effects of age of drinking onset and progression to drunkenness (i.e., time—in years—elapsed between the first contact with alcohol and the first episode of alcohol intoxication) on alcohol outcomes. In that study, we found that alcohol intoxication seems to kindle the expression of the vulnerability associated with early drinking onset, so that the early drinking onset promote the greater occurrence of alcohol-related negative consequences only among those who ever got drunk, while drunkenness naïve participants seem to be insensitive to the promoting effect of early-onset on alcohol consequences. That is, the age of drinking onset seems to be no longer relevant when there is no alcohol intoxication. Although more studies are needed, these results imply that reducing or impeding drunkenness is a valuable intervention milestone for those that have already begun to use alcohol.

Another relevant factor was the perceived frequency of alcohol use among same-sex peers. In general, a greater perception of peer drinking frequency increased the probability of belonging to the trajectories with more frequent HED. These results coincide with those found in adolescents (Chassin et al., 2002; van der Vorst et al., 2009; Danielsson et al., 2010) and emerging adults (Lee et al., 2012). It should be noted that the results suggest that the effect is specific for the drinking perception of same-sex peers. Previous studies have indicated that the strength of the association between perceived social norms and alcohol use depends on the specification and closeness of the reference group (Neighbors et al., 2010; Collins and Spelman, 2013). Usually, this association is stronger when the social norms derive from those close to the drinker (e.g., stronger with a best friend instead of the typical college student), compared to when the social norms derive from more distal social referents (Lewis and Neighbors, 2004; Collins and Spelman, 2013). Here, we found significant relations only when including perceived drinking norms of same sex-peers.

As in previous studies (Del Boca et al., 2004; Jackson et al., 2008; Ashenhurst et al., 2015), higher levels of sensation-seeking and positive urgency increased the probability of belonging to the trajectories with more frequent HED. These effects were small, which was not unexpected, given that distal variables tend to have smaller effects than proximal variables.

The *Moderate Decreasing Frequency* trajectory exhibited an intriguing pattern. The members of this trajectory drank, at the beginning of the study, similarly to those of the *Heavy Stable Frequency* and *Moderate Stable Frequency* trajectories. However, by the end of the study, they reduced their consumption to that exhibited by the *Stable Infrequent*, *Decreasing Infrequent*, and *No-HED* trajectories members. Interestingly, the only risk factor that allowed distinguishing between this trajectory and the *Heavy Stable Frequency* and *Moderate Stable Frequency* trajectories was alcohol intoxication. Thus, yet again lifetime drunkenness explains why, over time and despite exhibiting similar levels of alcohol use at the beginning, some participants kept stable levels of HED whereas others decreased this pattern of alcohol consumption.

A limitation of the study was the relatively high level of data attrition. Moreover, the drinking patterns of those who dropped out of the study were significantly different from those who kept participating. We can only speculate, yet it is possible that those who left the study may have endorsed trajectories with high HED frequency, had they continued participating. However, it is unlikely that this would have changed the quantity or the shape of the identified trajectories. Another limitation is the reliance on self-reported data, which might be affected by the participants’ ability to recall, which is sensible to telescoping/extrapolating biases. A non-probabilistic sampling was also used, thus limiting the possibility of generalizing the results to all Argentinean college students. The study also lacks the measurement of neurobiological characteristics. Recently, a preclinical study (Siciliano et al., 2019) showed that the activation of neurons projecting from the medial prefrontal cortex to the periaqueductal gray area predicted the emergence of risky alcohol drinking, after a phase of binge alcohol exposure. It has been also shown that some individuals are particularly sensitive to reward-associated cues, including those associated with alcohol (Versaggi et al., 2016). Hence, these individuals may be particularly prone to the effects of these stimuli on craving or relapse to drug-seeking behavior. Future extensions of the present study should consider adding these neurobiological measures, or appropriate proxies, to enhance the predictive ability of the model.

Despite these limitations, the study contributes towards identifying drinking trajectories among college students of an understudied sociocultural context. This work represents a step forward in the identification of risk factors differentiating between the HED trajectories. Although more research is still needed, the results found in this study suggest that alcohol intoxication is an important explanatory factor of alcohol consumption among Argentinean college students and a likely intervention target. Thus, the study has implications for the design of interventions aimed at detecting students at risk for engaging in problematic alcohol drinking. Such early detection at the individual level, which could also look for students featuring high levels of impulsivity and early age of first alcohol use, should be undertaken early in the student’s academic trajectory and complemented with campus-wide preventive actions, likely focusing in reducing normative perceptions on alcohol use.

## Data Availability Statement

The datasets generated for this study are available on request to the corresponding author.

## Ethics Statement

The studies involving human participants were reviewed and approved by the National University of Córdoba (Argentina) internal review board, and the protocol was reviewed by the National Agency for Promotion of Science and Technology of Argentina. The patients/participants provided their written informed consent to participate in this study.

## Author Contributions

The results presented are part of BV’s Ph.D. work. AP and RP designed the study and helped with data collection and supervised data analysis. BV organized the database, performed the statistical analysis, and wrote the first draft of the manuscript and subsequent versions. AP and RP edited the first draft of the manuscript and subsequent versions of it. All authors read, corrected, and approved the final submitted version.

## Conflict of Interest

The authors declare that the research was conducted in the absence of any commercial or financial relationships that could be construed as a potential conflict of interest.
